# Rare of Gaucher's Disease Complication of Splenic Multiple Gaucheroma

**DOI:** 10.2174/0118715303325860241023063241

**Published:** 2025-01-08

**Authors:** Nursima Çukadar, İrem Erdoğan Vezi̇roğlu, Canan Şehi̇t Kara, Ökkeş İbrahim Karahan, Tutkun Tali̇h, Fahri Bayram

**Affiliations:** 1 Department of Internal Medicine, Faculty of Medicine, Erciyes University, Kayseri, Turkey;; 2 Department of Endocrinology and Metabolic Diseases, Faculty of Medicine, Erciyes University, Kayseri, Turkey;; 3 Department of Radiology, Faculty of Medicine, Erciyes University, Kayseri, Turkey;; 4 Department of General Surgery, Faculty of Medicine, Erciyes University, Kayseri, Turkey

**Keywords:** Glucosylceramide accumulation, gaucheroma, quality of life, Gaucher’s disease, pseudotumors, enzyme replacement therapy

## Abstract

**Background:**

Gaucheromas, pseudotumors composed of Gaucher cells, are rare complications of Gaucher’s Disease (GD). They are usually seen in patients receiving enzyme replacement. Surgery is generally not recommended for these benign masses in treatment management. Most patients treated with Enzyme Replacement Therapy (ERT) show organ enlargement and improvement in laboratory values. In our case, no change in the size of the lesions was observed despite 4 years of standard dose ERT.

**Case presentation:**

A 27-year-old female, not having a known chronic disease and occasionally consulting doctors due to bone pain, weakness, and fatigue, visited the emergency department with the complaint of a nosebleed four years ago. The patient was found to have hepatosplenomegaly in physical examination and was referred to the hematology clinic because of pancytopenia. According to the physical examination and laboratory results, the desired leukocyte glucocerebrosidase level was 0.4 micromol/lt/hour (normal range > 2.5). Homozygous mutations of p.L483P and L483P were observed in genetic tests. Based on these results, the patient was diagnosed with GD. Although the patient received regular weekly treatment for 4 years, no significant change was observed in the spleen size. The patient was admitted to the hospital in April 2022 with complaints of abdominal fullness and indigestion. Her quality of life was deteriorating due to massive splenomegaly. A splenectomy was performed on the patient. In our case, splenic gaucheroma, a rare complication of Gaucher’s disease, was found to be present.

**Conclusion:**

These masses, which are a rare complication of GD, have started to be better recognized by radiologists and clinicians thanks to the case series shared, and it is clear that there is a need for standardization and further research in this field. With an increase in the number of cases and experiences in this field, there will inevitably be different and new developments in follow-up and treatment approaches.

## 1 INTRODUCTION

Gaucher’s disease is a rare autosomal recessive genetic disease caused by lysosomal Glucocerebrosidase (GBA1) enzyme deficiency [1]. Its worldwide incidence is about 1/40,000 to 1/60,000. It has a higher incidence in Ashkenazi Jews [2, 3]. Although there is no study on its prevalence in our country, the frequency is estimated to be above the general average due to the prevalence of consanguineous marriages.

Lysosomal glucocerebrosidase enzyme deficiency leads to the accumulation of the glycosphingolipid glucosylceramide, a component of cell membranes [1, 4]. This accumulation takes place in macrophages, which can be filled with glucosylceramide. Lipid-loaded macrophages (Gaucher cells) are mainly found in the liver, spleen, and bone marrow [1, 2].

The main clinical manifestations of the disease include hepatosplenomegaly, anemia, thrombocytopenia, leukopenia, bone pain, avascular bone necrosis, pathological fractures, and vertebral compression. The timing and pattern of symptoms are different for each affected individual [2]. GD is classically classified into three phenotypic variants. Type 1 is adult (non-neuropathic), type 2 is infantile (acute neuropathic), and type 3 is juvenile (subacute neuropathic). Type 1 Gaucher’s disease is the most common and clinically heterogeneous type. The most common clinical manifestations are pancytopenia, hepatosplenomegaly, bone pain, and bone pathologies. These diseases are diagnosed late, as they are rare, have heterogeneous multisystemic involvement, and show differences among their types [1, 2, 5].

The GBA1 gene is located on the long arm (1q21) of chromosome and contains 11 exons. Point mutation, inversion, deletion, and missense mutation can be seen in this gene region. The most common mutation is c.1226A>G (N370S). Other common mutations include c.84dup, c.1448T>C, and c.115+1G>A. Saposin C deficiency, which is a very rare condition, can also cause Gaucher’s disease. Saposin C is a glucocerebrosidase activator. Saposin C deficiency, which is characterized by a mutation in the PSAP (Prosaposin) gene, should be considered in patients with Gaucher’s disease, but with normal enzyme levels [6]

Gaucheroma is a rare complication of GD that clinicians do not recognize well. Gaucher cells accumulate focally in the liver, spleen, lymph nodes, and periosseous regions, forming benign lesions called gaucheromas. These typically progress slowly [7].

Deficiency of the lysosomal enzyme β-glucocerebrosidase and accumulation of its substrate in reticuloendothelial system cells in GD affect multiple organ systems. Lipid-laden macrophages transform into Gaucher cells, the pathognomonic feature of GD. Gaucheromas consist of cellular structures with the characteristics of macrophages that play an essential role in cancer-related inflammation. Generating non-classical CD16+/CCR4+ monocytes reflects the underlying cause of the accumulation of macrophages that can migrate to distant sites outside the reticuloendothelial system and lead to tumor-like gaucheromas [7, 8].

## 2 CASE PRESENTATION

### 2.1 Patient Information

A 27-year-old female patient was admitted to a physician clinic due to pain in the bones, fatigue, and a nosebleed four years ago. Afterward, she was referred to a hematology clinic for hepatosplenomegaly and pancytopenia (Table **1**). The bone marrow biopsy was performed, and it revealed hypercellular focal homogeneous bone marrow; CD68 and CD10 were positive in megakaryocytes and homogeneous in the existing material, with very dense histiocytes in the bone marrow. Dual-energy X-ray Absorptiometry (DEXA) was performed due to pain in the bones, and the lumbar total vertebral Z score was determined to be -3,6.

GD was suspected as a diagnosis in this patient *via* clinical and laboratory clues. Leukocyte glucocerebrosidase level was low, being 0.4 nmol/mL/hour (normal range > 2.5 nmol/ml/hour). Homozygous mutations of p.L483P and L483P were observed in the genetic analysis. Consequently, the patient was diagnosed with GD according to these results.

Her family history included consanguineous marriages among her parents, recurrent miscarriages of both her maternal and paternal grandmothers, and the loss of two siblings, with one having passed away at one day old and the other at five years old. The sibling who died at the age of five was suggested an operation because of splenomegaly, but the parents rejected the recommendation.

The imiglucerase enzyme replacement was administered at a dose of 60 units/kg every two weeks after the diagnosis. Although the patient received the treatment regularly for four years, no significant change was observed in the spleen size. Although pancytopenia was ameliorated, elevated ferritin levels remained (Table **1**).

### 2.2 Diagnosis

Although the patient received enzyme replacement weekly, she suffered from abdominal bloating and dyspepsia. Because of her massive splenomegaly, she had rapid satiety, bloating, and aesthetic concerns. She had a risk of spleen rupture secondary to a possible trauma due to her increased spleen size.

On the abdominal Magnetic Resonance Imaging (MRI), the liver size appeared to be 19 cm, and a complex cystic lesion (gaucheroma) with solid components was observed in the spleen with a size of 25x19x30 cm, which almost filled the left half of the abdomen, crossed the midline, and had a necrotic periphery with a large central area. No additional focus was observed in the liver, lung, and surrounding lymph tissues. MRI images of the patient are shown in Figs. (**1**) and (**2**).

### 2.3 Therapeutic Intervention

In light of the available information, the patient was evaluated by the multidisciplinary council, which included Erciyes University Faculty of Medicine, Endocrinology and Metabolism Diseases, Gastroenterology, Hematology, and General Surgery faculty members. Considering the risk of spontaneous splenic rupture and deterioration in quality of life, it was decided to perform a splenectomy. The splenectomy material is shown in Fig. (**3**).

**Fig. (4):**
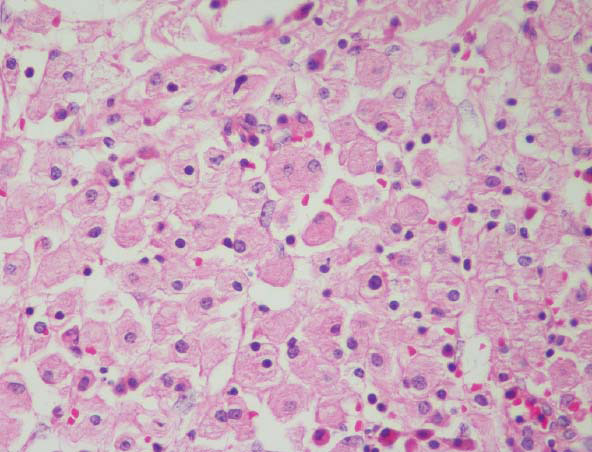
Microscopic images of gaucheroma.

In the pathological evaluation of splenectomy material, macroscopically, three beige nodular lesions were observed with a cystic appearance, weighing 9700 g and measuring 41 x 28 x 7.8 cm. These lesions had positive staining with CD68, KI-67 (1%), and S100 in the immunohistochemical examination. As a result, lesions were consistent with gaucheroma. In the light microscopic examination, the typical crumpled paper-like appearance formed by the accumulation of glucocerebroside in histiocytes at various magnification fields was observed (Fig. **4**).

### 2.4 Outcome and Follow-up

After the operation in May, the patient was followed up with a monthly hemogram and biochemistry (Table **1**), and radiological MRI follow-up was planned at 6-month intervals. The patient could easily participate in physical activities and was freed from the appearance of mass impairing the quality of life psychosocially and aesthetically, which has been dramatically improved.

## 3 DISCUSSION

GD is a rare genetic disease. GD is caused by a deficiency in the lysosomal enzyme beta-glucocerebrosidase. Deficiency of this enzyme leads to the accumulation of glycosphingolipids in macrophages and may induce oxidative stress by causing DNA damage in patients with GD. This causes cellular toxicity. Increased cytokine and chemokine levels are associated with macrophage activation and lead to impaired immune systems. T Follicular Helper (TFH) cells are a subset of helper T cells that play important roles in B cell follicles in the spleen and lymph nodes. TFH cells provide plasma and memory cells in the germinal center with assistance to B cells for maturation and differentiation, and allow customization of the antibody response. TFH biochemical and cellular changes in cells may contribute to the pathogenesis of GD [9, 10].

In the case of rare diseases, it is necessary to suspect the disease for diagnosis. Bone pain, fatigue, thrombocytopenia, splenomegaly, hepatomegaly, and low bone densitometry values (secondary osteoporosis) are among the events that should be considered in GD. In a suspected case, the first thing to do is to check the leukocyte glucocerebrosidase enzyme level in dry blood. GD patients have 10-15% fewer enzyme functions than usual. Mutation analysis of low-enzyme spread should be performed. An event in which a biallel pathological mutation is detected would confirm the diagnosis. If only one pathological mutation is detected in the mutation analysis and if there are no clinical symptoms, the patient could be a carrier; usually, no treatment is required. The spread of the disease may be a mutation that cannot be detected in usual tests, and further genetic analysis may be possible. The presence of Gaucher cells in a bone marrow biopsy is a critical finding for diagnosis, but it may not be necessary for most patients [2].

Gaucheromas, pseudotumors composed of Gaucher cells, are rare complications of GD. They are usually seen in patients receiving enzyme replacement [11]. Diagnosis is made due to the presence of cell cytoplasm with “wrinkled paper-like” features that define gaucheroma in biopsies taken from clustered lymph nodes in specific localizations, often in the abdomen. Gaucheroma was first described in 2002 by Lim *et al.,* as reported earlier in a study [12]. This first case, reported in a 13-year-old girl, was detected with a 5 cm mass in the mesentery approximately two years after the initiation of ERT. A surgical biopsy of the mass has been reported as characterized by a lymph node collection infiltrated with Gaucher cells at the base of the mesentery. In the cases of gaucheroma, as mentioned previously in the literature, the masses remain stable in size and biological behavior during follow-up. Surgery is generally not recommended for these benign masses. Kourla *et al.* recommended a combination of oral Substrate Reduction Therapy (SRT) or a higher dose of ERT for gaucheroma. However, some individual case reports have indicated that if a standard dose of ERT is maintained, an intermittent reduction in lesion size may occur [13]. Anemia may improve in the first six months of treatment in patients who are started on ERT therapy. The platelet response may vary depending on the extent of the splenomegaly. In moderate cases, organomegaly has been reported to resolve 12 to 24 months after the treatment [14]. Most patients undergoing ERT exhibit an improvement in organ growth and laboratory values. Although bone pain is reduced, recovery from skeletal changes is often delayed in this disease. However, intravenous ERT has been reported to significantly improve the quality of life [1, 2, 15]. ERT responses vary in patients with the same mutation and even in different organs [14]. Despite a 4-year standard dose of ERT in our case, no change was observed in the size of the lesions.

Complications are probably related to the timing of initiation of ERT; patients who begin treatment before splenectomy or the development of bone disease have a lower risk of complications. The precise location of remaining Gaucher cells and the associated risk of complications are unknown. In addition to chitotriosidase and other macrophage-derived cytokines, high ferritin levels are a feature of Gaucher’s disease. They indicate a response to therapy. Persistent hyperferritinemia represents abnormal iron metabolism and the presence of residual disease after years of intensive therapy. Iron overload is a major cause of morbidity and mortality. Residual toxic stores and abnormal metabolism of iron in Gaucher’s disease may contribute to an increased risk of malignancy [16].

In our case, there was pancytopenia at the time of diagnosis. Although pancytopenia improved after 4 years of enzyme replacement therapy, the patient had organomegaly that impaired her quality of life. The patient had difficulties in adapting to social life. In addition, high ferritin levels remained. After surgery, the patient was relieved of the psychosocial negative consequences caused by the mass image. In addition, the risk of cellular accumulation decreased with the regression of hyperferritinemia.

It should not be forgotten that gaucheromas, which should be considered when a mass image in the spleen or liver is encountered in patients with a diagnosis of GD, do not require surgery and do not change the treatment modality. One of the current imaging techniques, FibroScan^®^, can be used especially in the evaluation of liver gaucheromas [17].

## CONCLUSION

These masses, an infrequent complication of GD, are becoming more known by radiologists and clinicians thanks to the shared case series. In the literature review, it has been observed that questions, such as the prevalence of this disease, effects of treatments on gaucheromas, and their relationship with liver fibrosis-cirrhosis, remain unanswered [17]. There is a clear need for standardization in this area, and further research is needed. As the cases and experiences with this disease will increase, there will inevitably be new developments in follow-up and treatment approaches.

### Learning Points

- The gaucheroma does not always move uniformly and can occasionally be detected in multiple foci.
- Massive splenomegaly can be considered a splenectomy indication due to rupture risk secondary to trauma or impaired life quality in GD.
- The biopsy and surgical interventions should be administered when necessary, owing to the seeding possibility in GD.

### Patient Perspective

The patient can now easily participate in physical activities. She is happier that she is free from the mass that spoiled her appearance.

## Figures and Tables

**Fig. (1):**
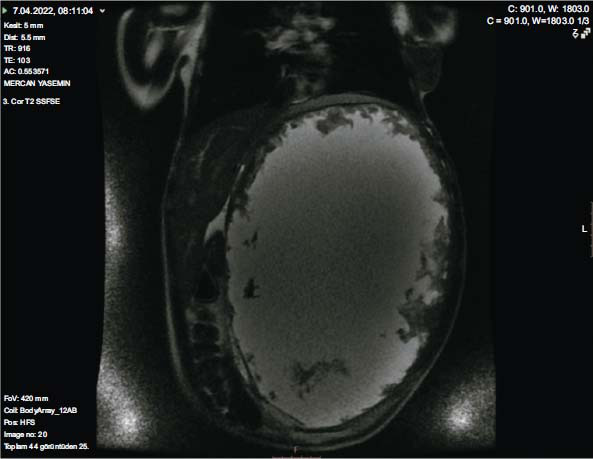
Heterogeneous hyperintense mass with solid (gaucheroma) areas filling the left half of the abdomen in the spleen site on T2-weighted coronal MR imaging.

**Fig. (2):**
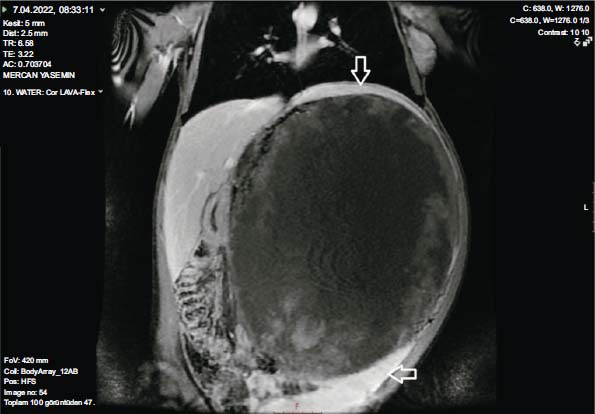
Complex cystic mass with solid (gaucheroma) areas filling the left half of the abdomen in the spleen site on T1-weighted contrast-enhanced coronal MR imaging. Parts of intact spleen tissue were also observed in the upper and lower poles.

**Fig. (3):**
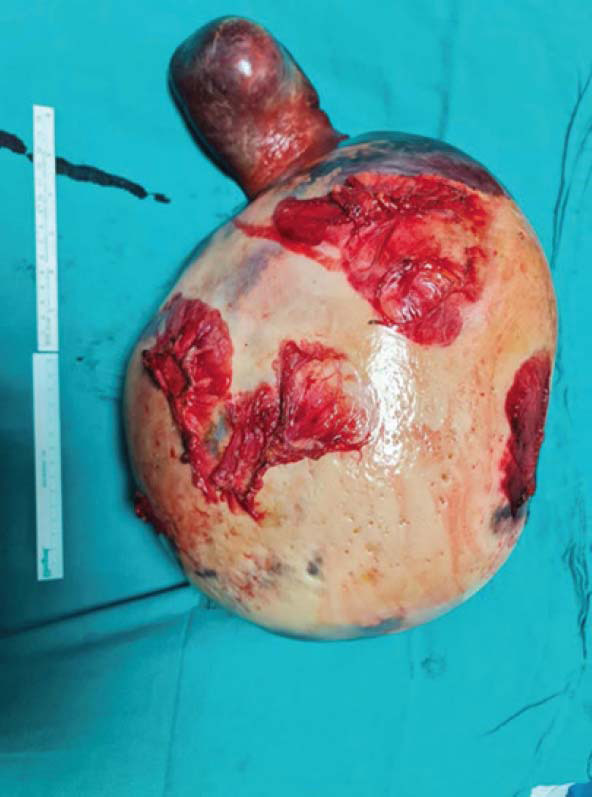
Macroscopic view of the spleen containing gaucheroma.

**Table 1:** The course of the patient's laboratory results.

Blood Parameters	Reference Ranges	Diagnosis Moment	After 4 Years of Treatment	Before Operation	3 Months After the Operation
WBC(10^3/µL) (10^9/L)	4.8-10.7	3.22	6.72	7.79	9.66
NEU(10^3/µL) (10^9/L)	2.2-4.8	1.53	4.38	4.84	4.47
MCV(FL)	81-99	69.2	73.4	73.9	71
HB(g/dL) (mmol/L)	12-16 (7.4-9.9)	8.1 (5.02)	11.9 (7.3)	12.5 (7.7)	14.1 (8.7)
PLT(10^3/µL) (10^9/L)	130-400	75	158	162	576
ALT(u/L) (µkat/L)	0-33 (0-0.55)	6 (0.10)	9 (0.15)	8 (0.13)	22 (0.36)
AST(u/L) (µkat/L)	0-32 (0-0.53)	17 (0.28)	11 (0.18)	11 (0.18)	15 (0.25)
Ferritin(ng/mL) (nmol/L)	15-150 (33.9-339)	721 (1633.7)	541 (1225.9)	429 (972.1)	31 (70.2)

**Abbreviations:** WBC: White blood cell; NEU: Neutrophil; MCV: Mean Corpuscular Volume; HB: Hemoglobin; PLT: Platelet; ALT: Alanine aminotransferase; AST: Aspartate aminotransferase; Ferritin: Ferritin.

## Data Availability

All data generated or analysed during this study are included in this published article.

## LIST OF ABBREVIATIONS

GO: Gaucher’s Disease
ERT: Enzyme Replacement Therapy
GBA1: Glucocerebrosidase
MRI: Magnetic Resonance Imaging

## STANDARDS OF REPORTING

CARE guidelines were followed.
